# The effect of mindfulness intervention on internet negative news perception and processing: An implicit and explicit approach

**DOI:** 10.3389/fpsyg.2023.1071078

**Published:** 2023-02-10

**Authors:** Ya Yang, Fang Su, Huan Liu, Xu Li

**Affiliations:** ^1^School of Journalism and Communication, Beijing Normal University, Beijing, China; ^2^Department of Communication, University of Macau, Macau, Macao SAR, China

**Keywords:** mindfulness intervention, global climate change, negative emotions, single category implicit association test (SC-IAT), online negative news

## Abstract

The internet facilitates the formation of the information society while also accelerating the viral spread of negative news and negative emotions, increasing public uncertainty and depression and impeding consensus, especially in the post-pandemic period. Mindfulness intervention, which has a positive effect on attention focus, self-regulation, and subjective wellbeing, is proven to mitigate negative emotional effects, and even alter mind patterns. The study aimed to give insight into the effect of mindfulness in the new media field, concerning trait mindfulness improvement, emotional arousal and regulation, and implicit attitudes from the perspective of intra-personal communication and positive communication. The study conducted a randomized pre-test–post-test control group design, with 3 (condition groups: mindfulness vs. placebo vs. control) × 2 (test times: pre vs. post). Participants who were exposed to negative news coverage with negative emotional arousal received 14 consecutive days of intervention. The results showed that mindfulness training can improve trait mindfulness effectively on the whole, especially in facets of describing, acting awareness, and non-judgment, and mitigate the negative effect from bad information coverage, while mindfulness intervention on mind patterns and expectations on controversial issues still awaited future empirical research. The present study intended to bridge the bonding between positive psychology and new media studies by focusing on individual attention improvement and negative emotion regulation, in the expectation that trait mindfulness can be beneficial in individual infodemic syndromes such as judgment bias and information exhaustion, and avoidance.

## Introduction

Social media has been credited and foreseen to build the public sphere and social good. While in recent years, it has been evident that the internet also facilitates the viral spread of negative news or bad (mis)information in everyday algorithm practice, this causes network anger and anxiety, forming information and belief echo chambers, impeding consensus, and increasing social polarization (Jamieson and Cappella, 2010; Thorson, 2013; Du and Gregory, 2017). The widespread massive information flow caused by the infodemic (PAHO, 2020) in the post-pandemic period exacerbates public uncertainty and depression (Zhang et al., 2020; Pine et al., 2021; Yoon et al., 2021).

Climate change has been a most pressing challenge faced by human society; data from 40 countries showed that social media was the main source and channel for the spread of (mis)information on climate change (Newman, 2020). In addition, new media platforms have done some crucial work on the production and reproduction of the meaning of climate change (Carvalho, 2010). A considerable quantity of online media coverage about this controversial issue can be seen around the globe, which has played the primary source of public exposure to information, and uncertainty and echo chamber effects in online discussions have been witnessed in various countries (Heal and Kriström, 2002; Williams et al., 2015).

Research showed that online news on controversial issues such as global climate change has strongly increased politicization and polarization and influenced public belief (Chinn et al., 2020). (Mis)information portrays visuals and texts that are most likely to influence individual emotions, where threats, danger, and doubts are implicated in eliciting negative emotions such as fear, anger, and exhaustion (Feldman and Hart, 2018; PAHO, 2020). It was further shown that negative emotions not only hook public attention (Zeng and Zhu, 2019) but also drive for virally dissemination and lead to the spiral of the evolvement of negative emotions in the network cycle, thus causing individual infodemic syndrome.

As a significant method in positive psychology, mindfulness intervention is proven to eliminate and control negative psychological conditions and mitigate negative impacts. Mindfulness usually refers to a training process of non-judgmental present-moment experience (Kabat-Zinn, 1990; Bishop et al., 2004), with open-minded acceptance of conscious thoughts, bodily feelings, and the environment (Hofmann et al., 2010; Poon and Jiang, 2020). High mindfulness is positively related to attention focus and reallocation (Lindsay and Creswell, 2017), stress alleviation and reduction (Grossman et al., 2004; Goldin and Gross, 2010; Xi et al., 2022), positive emotions and attitudes (Brown and Ryan, 2003; Hülsheger et al., 2013; Liu et al., 2022a), as well as subjective wellbeing (Brown and Ryan, 2003; Wallace and Shapiro, 2006; Liu et al., 2020, 2022b). The training breaks cognitive barriers, cultivate emotion regulation and satisfaction, copes with mental fatigue, and exalts current beliefs and future expectations (Langer, 2000; Hülsheger et al., 2013; Karelaia and Reb, 2015; Wang et al., 2019; Kudesia et al., 2022). In essence, mindfulness intervention altered the mind patterns from negative to positive, from closeness to openness and flexibility, and from path dependency to creation.

In recent years, empirical studies on mindfulness have flourished in cross-disciplinary research (Academic Mindfulness Interest Group [AMIG], 2006; Good et al., 2015), but the application of mindfulness intervention on infodemic syndrome, where negative emotions are induced by Internet bad information perception and processing, remains unknown. Thus, this study intended to extend the empirical perspective of mindfulness in the new media fields. It focused on the explicit and implicit attitudes changes associated with mindfulness-based stress reduction (MBSR) intervention, hypothesizing that it can reduce the negative effect and improve current tolerance and expectations by using reading materials of negative climate change coverage. Negative emotions and explicit attitudes were measured using questionnaires, and implicit attitudes were measured by behavioral experiments using the single category implicit association test (SC-IAT) cognitive paradigm.

## Materials and methods

### Participants

**FIGURE 1:**
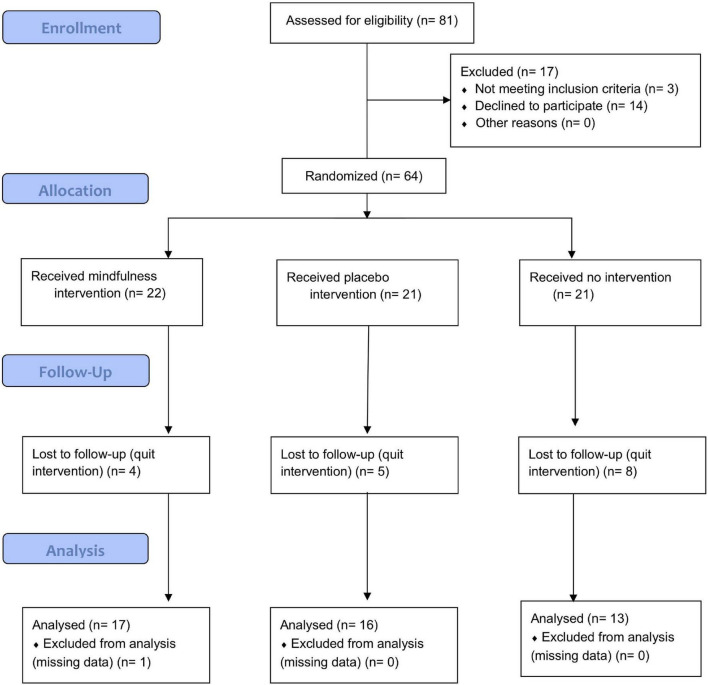
CONSORT flow diagram (Schulz et al., 2010).

The experiment initially recruited 81 non-psychology-major participants for mindfulness intervention and experiment. After the exclusion of three participants who failed to meet the previous criteria and 14 who declined successive time training, the study had 64 samples participating in the experiment. Among them, 46 effective participants completed the entire training and experiment, with 16 men and 30 women ranging in age from 18 to 45 years old (shown in Figure 1 and Table 1). The study applied G*power 3.1 (Faul et al., 2009) to carry out *post hoc* power analysis for the effective sample size (effect size *f*^2^ = 0.25, α = 0.05, 1-β = 0.918 > 0.8).

**TABLE 1:** Demographic variables of participants in three groups.

Variable	Classification	Group A (*N* = 17)		Group B (*N* = 16)		Group C (*N* = 13)	
		Total		Total		Total	
		*N*	%	*N*	%	*N*	%
Gender	Male	6	35.3	7	43.8	3	23.1
	Female	11	64.7	9	56.3	10	76.9
Age	15–18	1	5.9	0		0	
	19–23	15	88.2	5	31.3	10	76.9
	24–28	1	5.9	9	56.3	2	15.4
	29–35	0		2	12.5	0	
	36 or above	0		0		1	7.7
Education level	High school	0		1	6.3	2	15.4
	College or University	7	41.2	6	37.5	10	76.9
	Graduate School	10	58.8	9	56.3	1	7.7

All the participants in the training had no previous experience of regular training (no more than 1 h per week). They met the criteria of no chronic or acute mental or physical disease and no substance use or addictions. The participants were measured with moderate or low scores on the Positive and Negative Affect Scale (PANAS) before the experiment to ensure their normal emotion baselines. The experiment received the approval of the IRB in the Department, and the participants signed the informed consent before the experiment and got awarded after that.

### Procedures

The study conducted a randomized pre-test–post-test control group design, with 3 (condition groups: mindfulness vs. placebo vs. control) × 2 (test times: pre vs. post). For the three groups, participants first read the same stimulus reading materials, took the pre-test questionaries to mark the baseline of negative emotional condition, and then received the mindfulness/false/none training intervention for 14 consecutive days. After the training, the participants took the post-test questionaries to measure the emotional changes and the SC-IAT to assess the implicit attitudes of future expectancies for global climate change.

### Mindfulness training

The participants were randomly assigned to three groups, mindfulness training group, placebo group, and control group. In the mindfulness group, participants received successive 14-day professional MBSR training, 10 min per day in a fixed period. In the placebo group, participants received 14-day completely unrelated intonation practice; the time length was exactly the same as the training group to reduce the influence of the experiment itself. In the control group, the participants received no interventions.

The MBSR training was conducted online due to pandemic restrictions *via* the Tencent Meeting platform with all the participants’ face cameras on under their approval to make sure they were in an uninterrupted environment by themselves. The participants were separated into three online chat groups and were restricted from communicating with each other about the training. The three experimenters received professional training and consultancy from the professionals at the psychological counseling center.

In terms of the mindfulness intervention, the 14-day MBSR training paradigm was used in the study. In previous interdisciplinary studies, the short-period mindfulness training was 56, 30, 14, 10, or 7 days (Kral et al., 2018; Kudesia et al., 2022; Liu et al., 2022). The 14-day training was widely accepted and adopted to reduce social anxiety disorders (Papenfuss et al., 2022) and to enhance bonding through love, openness, and opportunity (Shreffler et al., 2019). The training continued for 10 min each day, with the introduction, breathing training, awareness mediation, body scanning, and the concluding section.

### Stimulus materials

Negative news coverage on global climate change and crisis served as emotional stimulus reading material in this study. The Depression-Anxiety-Stress Scale (DASS) was used for reading material measurement to ensure it would effectively arouse negative emotions. Four pieces adapted from original news were included to avoid single-material bias. The stimulus material was approximately 15,000 words in length, and it took the participants approximately 20 min to read the full text carefully. The same material was used in both the pre-test and post-test of the experiment.

Climate change or global warming issue has long encountered a sensitive “consensus gap” (Cook, 2016; van der Linden, 2022). In some social milieus, the public regard it as controversial or even skeptical of politically laden information dissemination, whereas in another social context, such as among the Chinese public, the situation is more widely accepted (Mou and Ke, 2022). However, there was bad information exploiting and linking it to fatalistic feelings, such as the above sea level or heat stroke caused by climate warming being the suggestion of humans being endangered, causing public fear, anxiety, and depression by repeatedly and virally spreading.

This type of information was referred to as negative news or bad information in the reading materials in this study. There were distinctions among fake news, including misinformation, disinformation, mal-information, and bad information. The first is unintentional false information. The latter three types contain deliberate manipulation, where mal-information is with real news but cherry-picking changes (Adams, 2022), and bad information refers to negative news with true information but harmful objectives (Wardle and Derakhshan, 2017). Bad information or negative news appeals to the uncertainty of the public, resulting in the illusory truth effect in the chain of repeating, rephrasing, and forwarding information (Wang et al., 2016) and even ethical and moral anxieties.

### Questionnaires

In the pre-test and post-test, the Five-Facet Mindfulness Questionnaire (FFMQ), the PANAS, and the Depression-Anxiety-Stress Scale (DASS-21) were applied.

First, trait mindfulness was assessed by the FFMQ, including 39 items representing five factors (Baer et al., 2006): observing items, acting aware items, describing items (such as putting thoughts, beliefs, and expectations into words), non-reacting items (such as watching feelings without getting confused), and non-judging items. A Likert scale ranging from 1 (rarely) to 5 (completely) was used to measure the degree of self-statement suitable to the participants’ conditions. The Chinese-adapted version of FFMQ (Deng et al., 2011) suggested satisfied validity and test–retest reliability (*r* = [0.44–0.74]). The internal consistency in this study was good (Cronbach’s α = 0.89).

Second, negative emotional arousal, the negative affect (NA) scale in PANAS (Watson et al., 1988) was used to evaluate subjective distress and negative emotions. The items contain negative adjectives, and a Likert scale ranging from 1 (very unlikely) to 5 (very strongly) was used to measure the extent to which the participants experienced the emotions described by these words. The Chinese-adapted version of PANAS (Qiu et al., 2008) suggested satisfied validity (Cronbach’s α = 0.89) and test–retest reliability (*r* = 0.47). The internal consistency in this study was good (Cronbach’s α = 0.87).

The DASS-21, which was compiled by the original DASS (Lovibond and Lovibond, 1995), kept the three dimensions of depression-anxiety-stress with seven items each while improving the efficiency of assessing related mood disorder symptoms (Crawford and Henry, 2003). The participants were asked to report how frequently the self-statements they experienced, including depression items (such as no longer having pleasant and comfortable feelings), anxiety items (such as feeling hand tremors), and stress (such as difficulty calming down), with a Likert scale ranging from 0 (none) to 3 (most frequently). The Chinese-adapted version of DASS-21 (Gong et al., 2010) suggested satisfied validity. The internal consistency in this study was good (Cronbach’s α = 0.95).

### SC-IAT procedure

The SC-IAT was designed to measure the implicit attitudes of a particular category (Karpinski and Steinman, 2006) and proved to have sufficient reliability in many fields (Bluemke and Friese, 2008; Stieger et al., 2010). The test procedure contained two tasks, compatible and incompatible, and four blocks, with 24 trials for practice and 72 trials for the formal test, respectively. Each trial contained 30 stimuli words (shown in Table 2).

**TABLE 2:** Experiment procedure of SC-IAT.

Block	Trials	Function	“F” key response	“J” key response
1	24	Practice	Positive words + climate word	Negative words
2	72	Test	Positive words + climate word	Negative words
3	24	Practice	Positive words	Negative words + climate word
4	72	Test	Positive words	Negative words + climate word

In the experiment, words related to global climate change issues were set as target words. There were 21 positive words (e.g., beautiful, optimistic, bright, joy, and happy) and 21 negative words (e.g., ugly, pessimistic, dim, anguish, and upset), which were selected from the Chinese Affective Words System (CAWS). Before the formal experiment, 30 participants were recruited to test the representativeness of 14 target words using a five-point Likert scale, and seven target words ranked top 50% of the total score (e.g., greenhouse, rains, dust, drought, typhoon, heat waves, and the hail). The words recruited in the test were all two-character Chinese words. Each trial contained a 500-ms cross-dot gaze, a 1,500-ms display of words, and an 800-ms empty screen.

In the SC-IAT, the positive association (“target word + positive word”) was in blocks 1 and 2, and the negative association (“target word + negative word”) was in blocks 3 and 4. In positive association tasks, a random sequence of the words and different response ratios of the two keys “F” and “J” on the keypad were applied, where target words, positive words, and negative words were presented in a 7:7:10 ratio so that 58% of correct responses were on the “F” key and 42% of correct responses were on the “J” key. Meanwhile, in the negative association tasks, the presentation ratio of target words, positive words, and negative words was 7:10:7, so 42% of correct answers were on the “F” key and 58% were on the “J” key.

## Results

Repeated-measures ANOVA with the test as a within-subject factor (pre vs. post) and the condition group (mindfulness vs. placebo vs. control) as the between-subject factor was conducted to predict the trait mindfulness and emotional statement. The trait mindfulness and emotional arousal scores (M ± SD) are shown in Table 3. The conditions in the pre-test of three groups were taken as the baseline under news reading stimuli.

**TABLE 3:** The trait mindfulness and emotional arousal scores in three condition groups (M ± SD).

	Mindfulness group (*n* = 17)		Placebo group (*n* = 16)		Control group (*n* = 13)	
	Pre	Post	Pre	Post	Pre	Post
Observing	26.94 ± 4.85	29.29 ± 4.74	28.56 ± 5.50	27.63 ± 4.30	26.31 ± 4.66	25.92 ± 5.62
Describing	26.59 ± 5.77	29.71 ± 3.62	28.31 ± 4.24	27.25 ± 4.65	25.38 ± 4.82	22.31 ± 4.70
Acting aware	27.47 ± 7.32	29.12 ± 4.09	25.19 ± 6.43	24.56 ± 7.58	24.00 ± 7.22	24.31 ± 7.51
Non-judging	22.59 ± 5.66	25.94 ± 3.42	20.13 ± 5.01	21.38 ± 4.30	20.85 ± 5.55	22.69 ± 4.55
Non-reacting	19.71 ± 3.02	23.65 ± 1.90	22.19 ± 4.12	22.50 ± 5.94	20.23 ± 4.30	20.77 ± 3.37
FFMQ total score	122.12 ± 14.74	137.71 ± 11.62	124.38 ± 16.63	123.31 ± 15.43	116.77 ± 18.97	116.00 ± 17.44
Negative affect	17.88 ± 4.64	15.06 ± 3.80	14.81 ± 5.11	18.00 ± 7.93	15.69 ± 5.59	16.92 ± 6.05
Stress	13.41 ± 5.00	10.47 ± 2.27	14.88 ± 5.14	15.50 ± 6.46	13.46 ± 4.75	13.62 ± 3.84
Anxiety	11.53 ± 4.09	10.35 ± 3.08	13.44 ± 5.10	13.38 ± 5.37	12.00 ± 4.43	11.23 ± 2.49
Depression	11.47 ± 3.86	9.24 ± 2.19	13.44 ± 5.34	13.38 ± 5.58	13.00 ± 5.20	13.00 ± 4.38
DASS total score	36.41 ± 12.08	30.47 ± 7.19	41.75 ± 14.71	42.25 ± 16.71	38.46 ± 13.45	37.85 ± 9.76

FFMQ, Five-Facet Mindfulness Questionnaire; negative affect was from The PANAS (Positive and Negative Affective Scale); stress, anxiety, and depression were from DASS-21 (Depression Anxiety Stress Scale).

### Trait mindfulness

**FIGURE 2:**
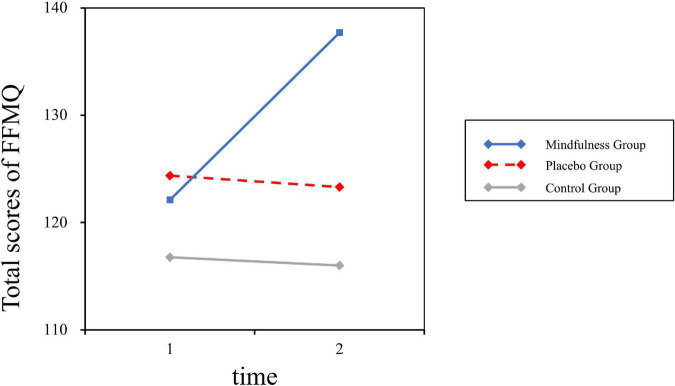
Interaction effect of group and time on the FFMQ.

The FFMQ results revealed that the impact of the 14 consecutive days of MBSR intervention was valid in the mindfulness training group. As is shown in Table 4, the main effect of the condition group was significant [*F* (2, 43) = 4.5, *p* = 0.02, η^2^*_*P*_* = 0.17], and the mindfulness group tended to have a higher score (*M* = 129.91, SD = 2.97) than the control group (*M* = 116.39, SD = 3.40, *p* < 0.001). In addition, there was a significant interaction effect between group and time [*F* (1, 43) = 3.73, *p* = 0.03, η^2^*_*P*_* = 0.15]; see Figure 2. The *post hoc* analysis revealed that after the mindfulness intervention, there were significant differences between the mindfulness group (*M* = 137.71, SD = 3.58) and the control group (*M* = 116.00, SD = 4.10, *p* < 0.001), as well as the placebo group (*M* = 123.31, SD = 3.69, *p* = 0.01). The main effect of time [*F* (1, 43) = 2.43, *p* = 0.13, η^2^*_*P*_* = 0.05] was not statistically significant.

**TABLE 4:** Statistical results of group and time in FFMQ, PANAS, and DASS-21.

	Main effect of condition group			Main effect of test time			Interaction effect of group × time		
	*F*(2, 43)	*p*	η^2^_*P*_	*F*(1, 43)	*p*	η^2^_*P*_	*F* (2,43)	*p*	η^2^_*P*_
Observing	1.10	0.34	0.05	0.18	0.68	0.00	1.66	0.20	0.07
Describing	5.99	0.01	0.22	0.15	0.70	0.00	4.48	0.02	0.17
Acting aware	3.53	0.04	0.14	0.09	0.76	0.00	0.23	0.80	0.01
Non-judging	3.69	0.03	0.15	6.72	0.01	0.14	1.14	0.33	0.05
Non-reacting	1.32	0.28	0.06	4.57	0.04	0.10	2.64	0.08	0.11
FFMQ total score	4.5	0.02	0.17	2.43	0.13	0.05	3.73	0.03	0.15
NA negative affect	0.00	0.99	0.00	0.40	0.53	0.01	4.80	0.01	0.18
Stress	3.65	0.03	0.15	0.55	0.46	0.01	1.41	0.25	0.06
Anxiety	2.20	0.12	0.09	0.84	0.37	0.01	0.21	0.81	0.01
Depression	4.65	0.02	0.18	0.61	0.44	0.01	0.60	0.56	0.03
DASS total score	2.06	0.14	0.09	3.21	0.08	0.07	3.36	0.04	0.14

FFMQ, Five-Facet Mindfulness Questionnaire; negative emotion (NA) was from PANAS (Positive and Negative Affective Scale); stress, anxiety, and depression were from DASS-21 (Depression-Anxiety-Stress Scale).

Furthermore, the findings of the FFMQ facets varied. First, observing facet, results showed that the interaction effect [*F* (2, 43) = 1.66, *p* = 0.20, η^2^*_*P*_* = 0.07], as well as the main effects of the group [*F* (2, 43) = 1.10, *p* = 0.34, η^2^*_*P*_* = 0.05] and time [*F* (1, 43) = 0.18, *p* = 0.68, η^2^*_*P*_* = 0.00], were not significant.

Second, describing facet, the main effect of the group was significant [*F* (2, 43) = 5.99, *p* = 0.01, η^2^*_*P*_* = 0.22]. The score was significantly higher (*p* = 0.003) in the mindfulness group (*M* = 23.85, SD = 1.01) than in the control group (*M* = 28.15, SD = 0.89). The main effect of time [*F* (1, 43) = 0.15, *p* = 0.70, η^2^*_*P*_* = 0.00] was not significant. The results revealed a significant interaction effect between group and time [*F* (2, 43) = 4.48, *p* = 0.02, η^2^*_*P*_* = 0.17], and the *post hoc* analysis indicated that the score of the post-test (*M* = 26.59, SD = 1.22) was significantly higher than the pre-tests (*M* = 29.71, SD = 1.05) in the mindfulness group (*p* = 0.03).

Third, acting with awareness, there was a significant difference between groups [*F* (2, 43) = 3.53, *p* = 0.04, η^2^*_*P*_* = 0.14], and the score in the mindfulness group was significantly higher (*M* = 28.29, SD = 1.13) than that in the control group (*M* = 24.15, SD = 1.30, *p* = 0.02) and the placebo group (*M* = 24.88, SD = 1.17, *p* = 0.04). The interaction effect [*F* (2, 43) = 0.23, *p* = 0.80, η^2^*_*P*_* = 0.01] and the main effect of time [*F* (1, 43) = 0.09, *p* = 0.76, η^2^*_*P*_* = 0.00] were statistically insignificant.

Fourth, the non-judging facet, the main effect of the group [*F* (2, 43) = 3.69, *p* = 0.03, η^2^*_*P*_* = 0.15] and time [*F* (1, 43) = 6.72, *p* = 0.01, η^2^*_*P*_* = 0.14] were both significant. The *post hoc* analysis revealed that the score in the mindfulness group (*M* = 23.68, SD = 0.76) was significantly larger than that in the placebo group (*M* = 20.75, SD = 0.79, *p* = 0.01). The post-test scored (*M* = 23.34, SD = 0.61) higher than that in the pre-test (*M* = 20.79, SD = 0.74). The interaction effect was not statistically significant [*F* (2, 43) = 1.14, *p* = 0.33, η^2^*_*P*_* = 0.05].

Finally, the facet of non-reactivity, the main effect of time [*F* (1, 43) = 4.57, *p* = 0.04, η^2^*_*P*_* = 0.10] was significant, and the score of post-tests (*M* = 22.31, SD = 0.61) was higher than that in the pre-test (*M* = 20.71, SD = 0.57). The main effect of the group [*F* (2, 43) = 1.32, *p* = 0.28, η^2^*_*P*_* = 0.06] and the interaction effect [*F* (2, 43) = 2.64, *p* = 0.08, η^2^*_*P*_* = 0.11] were statistically insignificant. Therefore, the effect of MBSR intervention on improving trait mindfulness was proved effective on the whole, especially in facets of describing, acting awareness, and non-judgment.

### Emotional arousal

As for the NA of PANAS, it was shown that the interaction effect of group and time was significant [*F* (2, 43) = 4.80, *p* = 0.01, η^2^*_*P*_* = 0.18]. The *post hoc* analysis indicated that in the mindfulness group, the negative affect in the post-test (*M* = 15.06, SD = 1.49) was significantly (*p* = 0.03) lower than that in the pre-test (*M* = 17.88, SD = 1.23), and comparatively, in the placebo group, the negative affect in the post-test (*M* = 18.00, SD = 1.53) was moderate significantly (*p* = 0.05) higher than in the pre-test (*M* = 14.81, SD = 1.27), which indicated the mindfulness training effectively reduced negative affect of bad information coverage.

**FIGURE 3:**
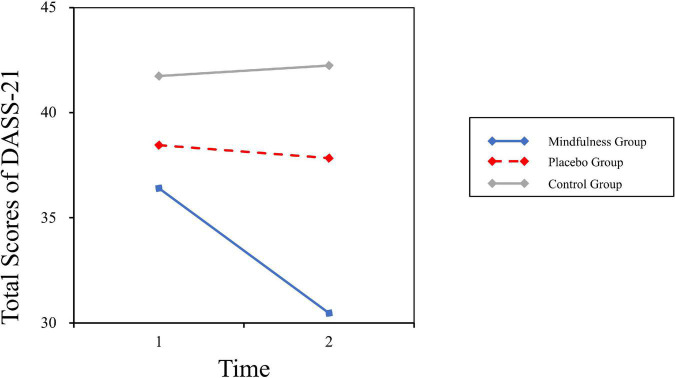
Interaction effect of group and time on DASS-21.

For DASS-21, the main effects of condition group [*F* (2, 43) = 2.06, *p* = 0.14, η^2^*_*P*_* = 0.09] and test time [*F* (1, 43) = 3.21, *p* = 0.08, η^2^*_*P*_* = 0.07] were not significant. The interaction effect was significant [*F* (2, 43) = 3.36, *p* = 0.04, η^2^*_*P*_* = 0.14], shown in Figure 3. The *post hoc* analysis showed that in the post-test, the score in the mindfulness group (*M* = 30.47, SD = 2.90) was significantly lower (*p* = 0.01) than that in the placebo group (*M* = 42.25, SD = 2.99). Furthermore, the score in the mindfulness group was significantly lower than that in the placebo group in both stress (*p* = 0.03) and depression evaluation (*p* = 0.02).

### Implicit attitudes

The primary IAT indicator is the *D*-score, which reflects the difference between the mean response times for compatible and incompatible trials (ranging from −2 to 2). It suggested that the more implicit associations one feels, the quicker categorizing of the compatible pairing (Greenwald et al., 2003). The SC-IAT results showed that the main effect of the condition group was not statistically significant [*F* (2, 43) = 0.30, *p* = 0.74, η^2^*_*P*_* = 0.01], while there was a higher *D*-score in the mindfulness group than that in other groups (mindfulness group 0.39 vs. placebo group 0.28 vs. control group 0.27). The higher *D*-score indicated better positive implicit attitudes in climate change expectations or full awareness and self-regulation for future preparation or confidence. However, it was not statistically significantly proven that mindfulness training altered mind patterns and cognitive bias from negative to positive.

## Conclusion

This study aimed to explore the effect of mindfulness in the new media field from the perspective of positive intra-personal communication by conducting a 14-consecutive-day mindfulness intervention experiment. The hypotheses that MBSR training can considerably improve mindfulness traits, such as attention focus and consciousnesses, as well as mitigate the negative effect of bad information coverage, were proven. While the following hypothesis, that mindfulness intervention can impose a positive impact on current tolerance and future expectations on controversial issues, was not significantly proven in the implicit attitudes test.

The study found that MBSR training improved trait mindfulness effectively in describing, acting aware, and non-judging factors. Furthermore, in congregant with the previous studies, the increase in trait mindfulness also facilitates decreasing subjective distress (Carmody et al., 2008; Shahar et al., 2010; Carpenter et al., 2019) and better recovery following initial reactivity to stressors (Fogarty et al., 2015). In the experiment, the mindfulness training group significantly reduced NA, which indicated that the MBSR intervention could decrease subjective distress and negative emotion caused by reading negative news on global climate change. The underlying mechanism can be that mindfulness practice increases individuals’ attention focus and awareness of their emotional states, thus improving attention control and switching, rumination, and emotion regulation (Chambers et al., 2008; Goldin and Gross, 2010; Hill and Updegraff, 2012).

## Discussion

To this end, mindfulness intervention has proved significant in the perspective of intra-personal communication and positive psychology. Previous studies demonstrated that mindfulness intervention has a potential role in enhancing positive emotions, such as happiness and meaningfulness, inner peace, and subjective wellbeing, such as self-compassion, awareness, self-efficacy, and other positive emotions (Liu et al., 2015, 2022a; Ivtzan et al., 2016). Nonetheless, individual and cultural habitus differences may moderate the effect. For instance, in Confucian culture, the value of introspective awareness, inner peace, and non-judging can be more attached than stimulus-driven pleasures (Wallace and Shapiro, 2006; Chiesa and Malinowski, 2011).

Given the reduced stress and depression after the MBSR training, this study found the practical implications of MBSR training in positive reappraisal and mental resilience in the media environment. Because FFMQ does not imply positive attitudes in general, this finding may help to dispel the misconception about mindfulness as a state of non-doing and relaxation (Huston et al., 2011). According to the broaden-and-build theory (Fredrickson, 2004), the state of mindfulness appears to have a positive valence impact on how we interpret our daily events and could be viewed as the initial phase in the reappraisal process (Garland et al., 2009).

In addition, mindfulness can work on individual mind patterns, which improves information exchange and communication modes between individual and external media environments. Effective intervention can alleviate the individual’s need for closeness and gain openness and innovation to the existing cognitive schema (Fiske and Linville, 1980), which was proved to be molded by media news frames and attributions (Entman, 1993; Pan and Kosicki, 2005). In the post-pandemic era, the public is exposed to massive (mis)information, overloaded with mostly negative information and emotion to perceive and process, causing individuals to lose awareness and judgment bias, leading to information exhaustion and even avoidance. The two components of trait mindfulness, self-regulation and present-moment consciousness (Bishop et al., 2004), can be beneficial in this perspective.

Furthermore, though the hypothesis of implicit attitudes toward the current tolerance and expectation of global climate change remained no different in this experiment, mindfulness training can be a dynamic cognitive process (Kabat-Zinn, 2003; Mikulas, 2011). In a recent study, the trait mindfulness significantly predicted less perceived severity and facilitated the participants to gain self-regulation and protection after the traumatic experience, showing positive confidence about post-pandemic healthy lives (Liu et al., 2022). MBSR intervention may have an influential impact on the not explicit but implicit attitudes toward negative information. Previous studies revealed that mindfulness training focused more on the present status, thus decreasing the capability of imaging the future possibilities and uncertainties in decision-making (Karelaia and Reb, 2015; Yuan et al., 2022) and does not influence rationalized knowledge hiding (Liu et al., 2022c), while other scholars argued that participants increased open-minded behaviors, creative thinking, performance, and metacognitive skills (Guo et al., 2018; Chen et al., 2022; Mitsea et al., 2022). In global climate change issues, in particular, connectedness with nature played a promising mediating role in mindfulness training and climate belief change (Wang et al., 2019). Thus, whether the extent to which MBSR training improved attention focus and regulation can influence attitudes and expectations over a longer period of intervention, such as lifetime self-training, or by a more complex moderation or mediation, such as social and nature connectedness, still awaits future empirical research.

## Limitations and implications

This leads us to the potential limitations and implications of this study. First, while the 14-day training modes successfully cultivate trait mindfulness, and previous studies have shown that there was no significant difference between single brief and long-term mindfulness interventions (Parsons et al., 2020), two facets observing and non-reacting remained unchanged; thus, further studies on 56-day training or longer are being considered. In addition, while online mindfulness and some mobile health applications have been shown to extend the effect in the positive intervention (Liu et al., 2022d), the restriction would be solved by repeated measures analysis, and further offline training exploration would be required.

Second, trait mindfulness, which was measured validly and reliably by FFMQ in previous and present studies, is proven different from state mindfulness, and evoking state mindfulness can predict and increase trait mindfulness in the trajectory of improving psychological positiveness (Vago and Silbersweig, 2012; Kiken et al., 2015), leading to a mediating effect design and test for further experiments.

Third, some studies have indicated the cognitive changes in brain signals such as N2 and P3 amplitudes, and amygdala activity during mindfulness meditation (Atchley et al., 2016; Kral et al., 2018; Klee et al., 2020), which also awaits subsequent cognitive neuroscience and communication research. Moreover, due to experimental methods and challenging differential attrition in the real-state psychological intervention (Graham and Donaldson, 1993), the external ecological validity of this study remained limited in explaining issues at the media and society levels. Meanwhile, second-generation interventions such as mandala art coloring games and loving-kindness meditation were as well proved to be effective (Liu et al., 2019; Xi et al., 2022) in mindfulness and anxiety reduction. Thus, the MBSR training paradigms need to improve in this permanent-online media society and post-truth infodemic communication environment.

## Data availability statement

The raw data supporting the conclusions of this article will be made available by the authors, without undue reservation.

## Ethics statement

The studies involving human participants were reviewed and approved by Ethics Review Committee at the School of Journalism and Communication, Beijing Normal University. The patients/participants provided their written informed consent to participate in this study.

## Author contributions

YY, HL, and XL designed the experiment. HL recruited the participants. XL worked on the reading materials. HL, FS, and XL conducted in the mindfulness training. FS collected and analyzed the scales and SC-IAT data. YY and FS wrote the manuscript. All authors participated in this study.
